# Environmental landscape and subsistence strategy of the Shunshanji Culture: A review

**DOI:** 10.3389/fpls.2022.966635

**Published:** 2022-07-22

**Authors:** Zhenwei Qiu, Huiyun Rao

**Affiliations:** ^1^National Museum of China, Beijing, China; ^2^Key Laboratory of Vertebrate Evolution and Human Origins, Institute of Vertebrate Paleontology and Paleoanthropology, Chinese Academy of Sciences, Beijing, China; ^3^Center for Excellence in Life and Paleoenvironment, Chinese Academy of Sciences, Beijing, China

**Keywords:** Shunshanji cultural site group, vegetation landscape, landform, subsistence strategy, paddy field cultivation system

## Abstract

The Shunshanji Culture is the earliest known Neolithic culture in the mid-lower Huai River. In recent years, with new discoveries and deeper studies of the Shunshanji Culture, the concept of the Shunshanji cultural site group has gradually formed. Among them, various types of rice remains have been unearthed in large quantities, which provide key materials for discussing rice farming, rice cultivation and domestication, and related issues in the Huai River Basin. Previous studies have conducted reconstruction of local vegetation landscape and analysis of subsistence strategies on some systematically excavated Shunshanji cultural sites and obtained some new understandings. Integrative research, however, is lacking. In this review, we combine the construction of the local environmental landscape with the settlement landform within the Shunshanji cultural site group and then incorporate it into the regional environmental evolution of the mid-lower Huai River. The consistency and difference in their subsistence were also summarized. In particular, we focus on the relevant clues of the early paddy field cultivation system in the region and perform comparative studies.

## Introduction

**Figure 1:**
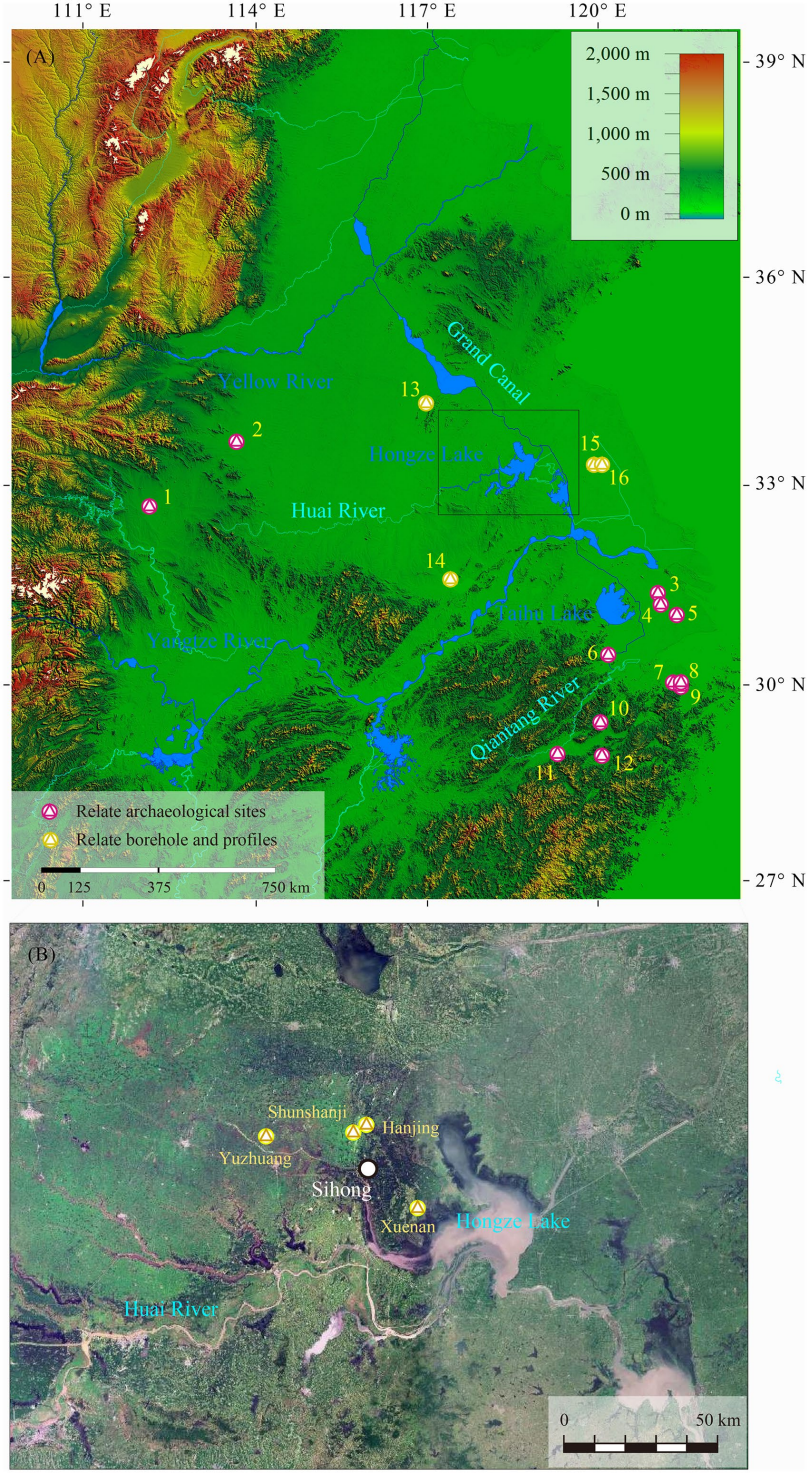
Study area **(A)** and an enlarged view of the distribution area of the Shunshanji cultural site group **(B)**. 1. Baligang site; 2. Jiahu site; 3. Zhumucun site; 4. Jiangli site; 5. Guangfulin site; 6. Maoshan site; 7. Shi’ao site; 8. Tianluoshan site; 9. Hemudu site; 10. Shangshan site; 11. Hehuashan site; 12. Huxi site; 13. Huangkou borehole; 14. Chao Lake borehole; 15. Sanchakou profile; 16. Qingfeng profile.

The Huai River Basin was located in the climatic and geographical transition zone, which directly influences and reflects its cultural tradition, making it an important cultural corridor (Guo, 2012). The early Holocene in the Huai River Basin corresponds to the early and middle Neolithic periods. There is no clear clue about the regional remains of the early Neolithic Age, and the Middle Neolithic (9,000–7,000 BP) remains can be roughly divided into three small regional systems (Zhang et al., 2018), among which the Shunshanji Culture (8,500–8,000 BP; Nanjing Museum and Sihong Museum, 2016; Lin, 2017; Yan, 2018; Zhu, 2018; Qin, 2021) is mainly distributed in the north of the mid-lower Huai River. The Shunshanji cultural remains were first discovered in the 1960s (Yin and Zhang, 1964), and after several excavations since 2010, more sites with similar cultural features were discovered and excavated on the west side of Hongze Lake in the past decade. With the corresponding research deepened, the concept of the Shunshanji cultural site group has gradually formed. The sites are mainly distributed in the middle and lower reaches of the Huai River, especially in the west of the Hongze Lake, and four sites have been discovered so far, including Shunshanji, Hanjing, Xuenan, and Yuzhuang (Figure 1). Among these, excavations were carried out in the former three sites, and a full survey was conducted at the last one. In addition, although some nearby sites have unearthed cultural remains with similar characteristics of the Shunshanji Culture, they generally show cultural and locality differences and were excluded from the group.

Discussions on the local environmental landscape and the economic pattern of settlements have been conducted on the sites of Shunshanji (Wu et al., 2017; Luo et al., 2021), Hanjing (Qiu et al., 2018b, 2022) and Xuenan (Qiu et al., 2021a). Carbonized rice, domesticated rice spikelets and domesticated rice phytoliths were unearthed from all the three sites of the Shunshanji Culture, indicating that rice was planted 8,000 years ago in the middle and lower reaches of the Huai River. Combining the 8,000-year-old rice (Zhang and Hung, 2013) excavated from the Jiahu site (Cheng, 2016) in the upper reaches of the Huai River and the Baligang site (Deng and Gao, 2012; Deng et al., 2015) near the Han River Basin, it was indicated that the Huai River Basin played a very important role in the process of rice domestication in prehistoric China. This new deduction challenged the general understanding that rice farming originated in the mid-lower reaches of the Yangtze River at approximately 10,000–9,000 BP in China (Yan, 1982; Zhao, 2009).

In the early Holocene, from the mid-lower reaches of the Yangtze River to the lower reaches of the Yellow River, the expansion of rice consumption or the early spread of rice farming may be related to the climatic environment, cultural and technological transmission, and human migration during the Holocene Optimum (Qin, 2012; Zhang and Hung, 2013). Within a specific spatiotemporal profile, the origin or development of rice farming may be more restricted by the local vegetation landscape, micro-geomorphic environment and other factors.

In this paper, based on the regional environmental background, the micro-geomorphology of the Shunshanji cultural settlement was investigated. Then the results of researches on the livelihood pattern of the Shunshanji Culture were integrated. In particular, the rice cultivation behavior and domestication process during the Shunshanji cultural period were discussed.

## Regional landscape and landform of the Shunshanji Culture

### Early Holocene environmental background recorded by natural sedimentation in the mid-lower Huai River

Palynological analysis has revealed the regional natural environmental landscape and climatic characteristics. Previous study on the Huangkou borehole in Xiao County, Anhui Province, revealed that the regional vegetation was coniferous and broad-leaved mixed forest, and the local vegetation was meadow composed of *Artemisia* and *Chenopodium* (Huang et al., 2005). Similar work on Qingfeng and Sanchakou profiles in Jianhu County, Jiangsu Province, revealed a mixed evergreen and deciduous broad-leaved forest with coniferous forest and halophytic meadow vegetation, and the representative plants included arboreal species of *Quercus*, *Castanea*, *Cyclobalanopsis*, *Castanopsis*, *Pinus* and herbs of Chenopodiaceae, Ranunculaceae, Poaceae and *Artemisia* (Zhao et al., 1990; Tang et al., 1991, 1993; Wu et al., 1996; Wu, 2018). The paleoenvironmental reconstruction of the Chao Lake, which is close to the geographical distribution of the Shunshanji Culture, reached the following conclusions: (1) The regional vegetation landscape was evergreen and deciduous broad-leaved mixed forest from 9,870 BP to 7,700 BP, which was in the rising stage of temperature fluctuation in the early Holocene; (2) At approximately 7,700 BP, the palynological assemblages changed with increased terrestrial herbs and shrubs and decreased deciduous trees, and the surrounding area of the Chao Lake probably experienced a high temperature and drought climate (Wang et al., 2008). This abrupt environmental event corresponds to the short drought period of 8,000–7,700 BP recorded in the Bangong Co (Gasse et al., 1996) and SumxiCo (Van Campo and Gasse, 1993) profiles in the western Tibetan Plateau. What was more, the climate change in northwest India also happened at the same period (Gasse et al., 1996). All these contemporary environmental events may be related to the global climate change caused by the sudden weakening of the Asian monsoon at approximately 8,000 BP, which is reflected in the δ^18^O records of high-resolution stalagmites in Sanbao Cave (Shao et al., 2006), Dongge Cave (Dykoski et al., 2005), and Qunf Cave (Fleitmann et al., 2003). In conclusion, during the early Holocene (10,000–7,500 BP), the mid-lower reaches of the Huai River generally presented a coniferous broad-leaved mixed forest and grassland type vegetation assemblage, with wetland vegetation such as reed and cattail mostly distributed, while the climate was cool and wet.

According to Yin (2018), the GX2 profile at Jianghu recorded a shallow sea environment (tidal inshore area) during ca. 8,500–7,500 BP (equivalent to the Shunshanji Culture). In the early Holocene, sea water invaded the Xuyi and Gaoyou areas many times which were close to the Shunshanji cultural distribution area on the west bank of the Hongze Lake; In the Middle Holocene, the relatively stable bay lagoons gradually formed the Jianghuai water network, including the prototype of present day Gaoyou Lake (Wu, 2018; Yin, 2018). The Hongze Lake was derived from an artificial water conservancy project named as “to preserve and clean up the Yellow River” after the Ming Dynasty. It is worth noting that the earliest archeological sites ever found in the Jianghuai area to the east of the Hongze Lake were dated back to 7,000 BP, which can be well fitted with the gradual retreat of the coastline in eastern China since 7,000 BP revealed by paleoenvironmental sediment indicators (Wu, 1990; Zhu et al., 1996; Xue et al., 2010; Yin, 2018). Therefore, the relatively concentrated distribution of the Shunshanji cultural sites in the western Hongze Lake is likely to reveal a larger transgression range in the early Holocene. However, as the elevation of the Hongze lakebed is over 10 meters above sea level, more direct evidence is needed to confirm this hypothesis.

### Landform of Shunshanji cultural settlement

The Shunshanji site (33°34′34.23″ N, 118°10′11.44″ E, 26–30 meters above sea level) is on sloping land on the north side of the Chonggang Hill, with a drop of 4 meters from the northeast to the southwest, and covers an area of approximately 175,000 square meters (Nanjing Museum and Sihong Museum, 2016). The Hanjing site (33°35′ 27.42″ N, 118°13′3.18″ E, 20 meters above sea level) is also located at the front of the hillside on the north side of the Chonggang Hill, which is approximately 4 kilometers west of the Shunshanji site with an area of approximately 50,000 square meters (National Museum of China et al., 2018). The Chonggang Hill is a long and narrow uplift in the north–south direction, and the highest point is approximately 60 meters above sea level. The Chonggang Hill is surrounded by flat plains with an altitude of 16–18 meters. The Xuenan site (33°20′39.00″ N, 118°24′43.56″ E, 20 meters above sea level) is 45 kilometers away from the Hanjing site in the northwest and adjacent to Hongze Lake in the east. The site is mainly located at a relatively high-pitched position in the area with an unknown site area where the topsoil is now farmland (Qiu et al., 2021b). The Yuzhuang site (33°33′20.91″ N, 117°51′34.03″ E, 20–30 meters above sea level) is located on the piedmont slopes of the southwest foothills of the Ping Hill, approximately 30 kilometers east of the Shunshanji site, with an area of approximately 15,000 square meters (Zhang et al., 2018).

**Figure 2:**
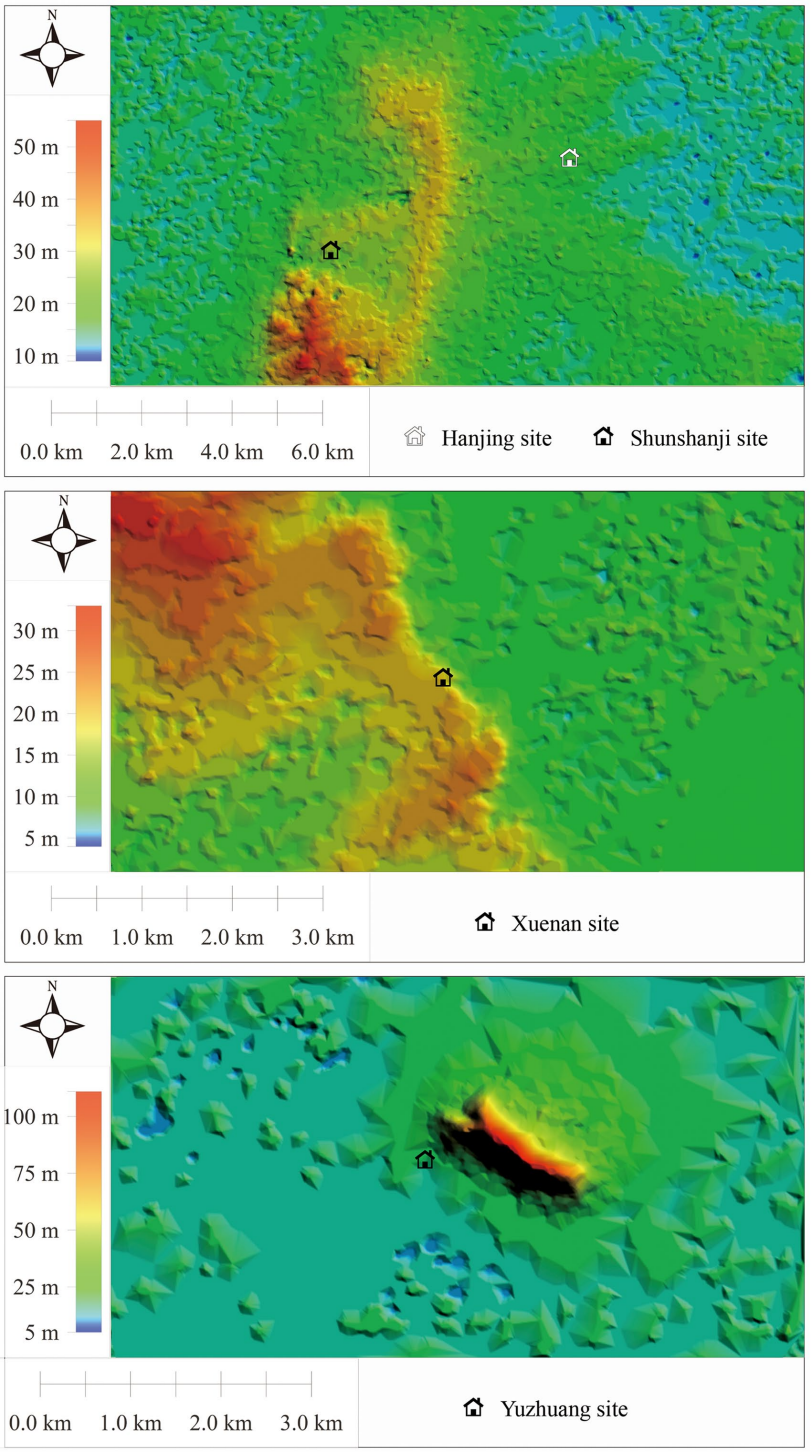
Elevation grid of the Shunshanji cultural settlements.

Through the simulation and reconstruction of the elevation grid (Figure 2), the micro-geomorphic characteristics of the Shunshanji cultural settlements can be summarized as follows. Firstly, these sites are generally located in the edge zone extending from hill to plain or basin, and most of them are moat settlements. Secondly, according to the historical records of the Huai River, flood and sea level changes are likely to have a great influence on regional landforms, especially represented by the formation of the Hongze Lake (Wu, 2018; Yin, 2018). The landform during the burial process of the Xuenan site should vary widely from its native landscape. In addition, due to the strong influence of the flooding of the Huai River and the role of the Hongze Lake, it is difficult to know accurately about the ruins of the native landscape by drilling. Thirdly, the area of the sites varies greatly, from more than 10,000 square meters to more than 100,000 square meters. On the one hand, it should be limited by the microscopic geomorphic environment where the sites are located. On the other hand, it may also reflect the difference in the settlement functions and layouts, especially between the closely located Shunshanji and Hanjing sites.

The site landform is in the process of continuous epigenetic changes (Wang, 2017). The different roles of accumulation and erosion processes, human activities and other factors in the micro-geomorphic landforms of the Shunshanji cultural site group should be fully considered. In particular, the corresponding geomorphic environmental factors should be focused.

## Local landscape and subsistence strategy of the Shunshanji Culture

A comparative study indicated that the study of cultural accumulation in archeological sites is effective in reconstructing the local environmental landscape (Qiu et al., 2016, 2020). In addition, animal remains unearthed at the site are also direct evidences for studying the surroundings and economic patterns at that time. Because some wild animals are sensitive to the natural environment, their existences can help us rebuild the local environment to a certain extent. For example, *Cervus nippon* generally lives in thickets or grasslands at forest edges, *Elaphurus davidianus* mostly inhabits swamp grassland or reed land on plains, and *Hydropotes inermis* and other small deer mostly live in reed or thatched environments near rivers, lakes, and beaches, while wild boar inhabits dense bushes, wet deciduous forests or grasslands (Sheng et al., 1999).

Plant remains analyses revealed that the vegetation types of the Shunshanji site mainly include reed, rice, barnyard grass, Cyperaceae and other aquatic or wet plants, together with oak, job’s-tears, Cucurbitaceae, Portulacaceae, Chenopodiaceae, Asteraceae, Poaceae and other terrestrial plants (Table 1). Among them, rice showed the characteristics of early domestication, and the degree of domestication gradually increased from the first to the third stage of the Shunshanji Culture (Luo et al., 2016).

**Table 1:** Plant assemblages of the Shunshanji site.

Analytical methods	Plant taxa	Remarks and references
Flotation	*Oryza sativa*, Portulacaceae, *Rumex*, Chenopodiaceae, *Mollugo stricta*, Asteraceae, etc.	(Zhang et al., 2014)
Phytolith analysis	*Oryza sativa*, *Phragmites australis*, *Echinochloa crusgalli*, Cyperaceae, Panicoideae, Pooideae, etc.	(Zhang et al., 2014; Luo et al., 2016; Wu et al., 2017)
Starch grain analysis	*Coix lacryma-jobi*, Triticeae, *Oryza sativa*, *Trichosanthes kirilowii*, *Quercus*, etc.	Samples from stone tools and pottery caldrons (Zhang et al., 2014; Yang et al., 2016)

Zooarchaeological research (Nanjing Museum and Sihong Museum, 2016) revealed the fauna of the Shunshanji site as follows: (1) At the first stage of the Shunshanji Culture, Suidae and large Cervidae were mainly discovered, with the former dominant; (2) At the second stage of the Shunshanji Culture, the fauna mostly included Suidae, Cervidae, *Bubalus*, *Canis*, Felidae, and Testudines, among which Suidae species account for the largest proportion; and (3) At the third stage, Suidae, large and medium Cervidae, *Canis* and *Panthera tigris* were reported. Among them, the dogs have been domesticated. As to the family Suidae, wild pigs are predominant along with a certain proportion of domestic pigs holding early domestication characteristics. Thus the ancestors of the Shunshanji site mainly obtained meat resources through hunting (wild pig and deer), supplemented by livestock breeding.

**Figure 3:**
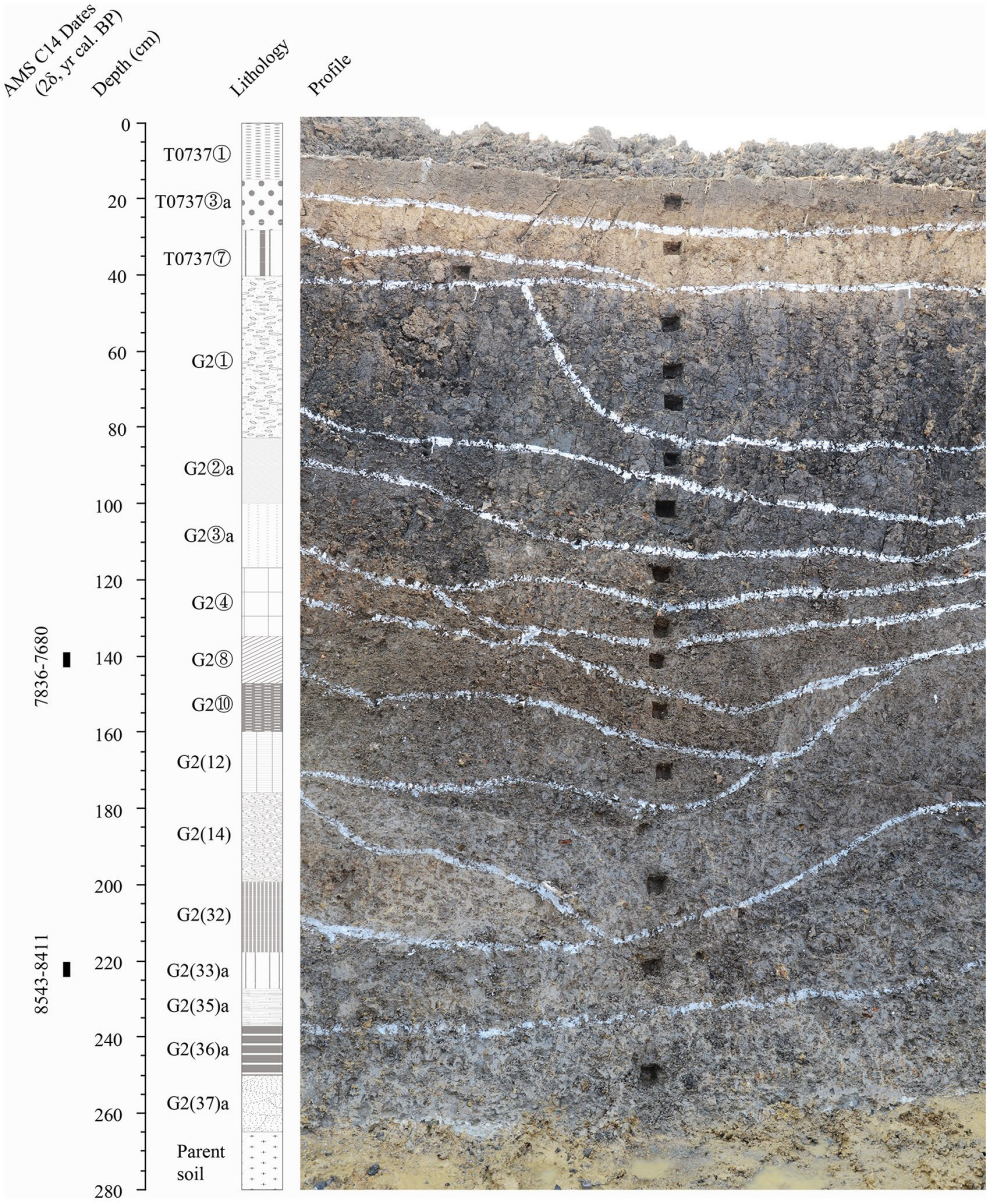
Sampling profile of the Xuenan site for analyses of pollen and phytolith.

Analyses of plant remains at the Hanjing site revealed that rice cultivation and wild plant resource collection coexisted for a long time; Among the unearthed animal remains, pigs were the most abundant mammals, followed by deer, and there were also many freshwater animals, mainly including fish and turtles (Qiu et al., 2018b). Even though few plant remains were unearthed from the accumulations in the trenches (relatively less affected by human activities at that time, Figure 3) at the Xuenan site, various types of plant utilization were still noticed. In addition to rice production, wild plant resources were also consumed by the ancestors, including *Euryale ferox* and other tubers (Qiu et al., 2021a). In addition, many mussel shells, mussel tools, fish and turtle bones, and mammal bones were also unearthed at the Xuenan site, reflecting the effective use of aquatic and terrestrial animal resources and even domestication of pigs (Qiu et al., 2021b).

Besides those evidences mentioned above, the findings and study of tools and vessels from the Shunshanji Culture (Nanjing Museum and Sihong Museum, 2016; National Museum of China et al., 2018) could also be integrated to shed light on a comprehensive understanding of its subsistence strategy. The stone tools mainly include axe, adze, millstone, and grindstone while cone dominates bone and horn artifacts. But no explicit farming tools were discovered. The majority of the pottery was made of sand-tempered or plant-tempered pastes, or fine clay pastes. The typical pottery assemblages consisted of *fu*-cauldron, jar, *bo*-bowl, bowl, ring footed plate, vessel lid, cup, bottle, foot supporter, spinning wheel, and file, of which the *fu*-cauldron and the foot support were used as a cooking set. Most of the *fu*-cauldron vessels have a black inner surface and a reddish or reddish-brown outer surface, indicating that they were indeed used for cooking or similar heating activities. Analysis of organic residues from potshards suggests the pottery was mainly used to process C3 plants, aquatic foods and terrestrial non-ruminant animals (Qiu et al., 2022; unpublished data from lipid analysis of pottery residues conducted by Rao).

On the whole, multiple indicators, such as carbonized plant remains, phytoliths and starch grains (Luo et al., 2016; Yang et al., 2016; Wu et al., 2017), suggest that gathering was the main way for the ancestors to obtain plant food resources (*Quercus*, *Trichosanthes kirilowii*, *Coix lacryma-jobi*, etc.) at the Shunshanji site while rice production was an effective supplement. Similar subsistence strategy was also found in the nearby Hanjing and Xuenan sites (Qiu et al., 2021a, 2022), and the latter also showed obvious intake of seafood (unpublished data from lipid analysis of pottery residues conducted by Rao). This may reflect the role of human selection superimposed by microscopic geography and environmental landscape. Whether this phenomenon has universality and timeliness in the mid-lower Huai River or even the lower reaches of the Yangtze River deserves special attention and further exploration.

Pollen analysis (Qiu et al., 2021a) showed that during the Shunshanji Culture, the evergreen-deciduous broad-leaved forest around the Xuenan site tended to decrease, and terrestrial herbs represented by Poaceae developed (see Figure 3; Table 2 for detailed sampling section and lithology description). *Setaria*, *Digitaria*, *Artemisia*, *Plantago*, Chenopodiaceae and other plants grew on the forest edges or wastelands; Cyperaceae, reed, rice and other plants grew in wetlands; and *E. ferox*, *Typha* and *Myriophyllum* were distributed in lakes and swamps. The water area expanded, water activities intensified, and the climate was generally warm and wet with fluctuations, presenting a wetland landscape suitable for the development of rice agriculture. Meanwhile, the fauna of the Shunshanji Culture generally indicated a coexistence of forest, grasslands and wetlands in the area where the sites were located.

**Table 2:** Lithology description of the section at the Xuenan site where pollen and phytolith analyses were carried out.

Cultural attributes	Deposit	Lithology description			
		Color	Structure	Texture	Inclusions
Modern	T0737①	gray	hard-close	clay	modern plant roots
Shunshanji Phase 3	T0737③a	light gray	loose	clay	sintering red soil
	T0737⑦	dark gray	hard-close	clay	potshards, animal bones, shells
	G2①	black	loose	clay	potshards, sintering red soil, animal bones, shells
	G2②a	black gray	hard-close	clay	animal bones, shells
Shunshanji Phases 1&2	G2③a	black gray	hard-close	clay	potshards, sintering red soil, animal bones, shells
	G2④	dark gray	hard-close	clay	potshards, stone tools, sintering red soil, animal bones, shells
	G2⑧	dark gray	loose	clay	potshards, animal bones, shells
	G2(10)	gray	hard-close	clay	potshards, sintering red soil, animal bones, shells
	G2(12)	gray	loose	clay	potshards, sintering red soil, animal bones, shells
	G2(14)	light gray	hard-close	clay	potshards, animal bones, shells
	G2(32)	dark gray	hard-close	clay	potshards, animal bones, shells
	G2(33)a	black gray	hard-close	clay	potshards, animal bones, shells
	G2(35)a	light gray	hard-close	clay	potshards, animal bones, shells
	G2(36)a	black gray	loose	clay	potshards, animal bones, shells
	G2(37)a	light gray	loose	clay	potshards, animal bones, shells
	Parent soil	yellowish	hard-close	clay	

In summary, during the Shunshanji Culture, the climate in the mid-lower Huai River was warm and humid, and the ancestors lived in a landscape surrounded by trees, shrubs, grasses and wetlands (Table 3), showing a subsistence strategy dominated by hunter-gathering supplemented by livestock raising and rice cultivation. Moreover, there are certain differences between sites.

**Table 3:** Main local vegetation landscape of the Shunshanji cultural site group.

Plant type	Plant taxa
Arboreal	*Quercus*, *Castanopsis*, *Ulmus*, Fagaceae, *Betula*, *Corylus*, *Carpinus*, Lauraceae
Shrubs and terrestrial herbs	*Rumex*, *Mollugo stricta*, *Artemisia*, *Plantago*, *Setaria viridis*, *Digitaria sanguinalis*, *Digitaria chrysoblephara*, *Physalis alkekengi*, *Patrinia scabiosaefolia*, *Galium aparine* var. *echinospermum*, *Vicia sepium*, *Coix lacryma-jobi*, *Trichosanthes kirilowii*, Triticeae, Panicoideae, Pooideae, Poaceae, Chenopodiaceae, Bambusoideae, Portulacaceae, Chenopodiaceae, Asteraceae, Amaranthaceae, Caryophyllaceae, Fabaceae
Wet herbs	*Oryza sativa*, *Phragmites australis*, *Echinochloa crusgalli*, Cyperaceae
Aquatic herbs	*Euryale ferox*, *Myriophyllum*, *Juncellus serotinus*, *Typha*
Ferns	*Ceratopteris*, Triletes, Monoletes

It should be noted that the Jiahu site was located in the upper reaches of the Huai River during the Jiahu Culture, whose local vegetation landscape was as follows (HPICRA, 1999): (1) There were sparse deciduous broad-leaved forests consisting of *Quercus*, *Castanea*, *Juglans*, *Corylus* on the nearby hillsides, and *Ulmus*, *Salix*, *Morus*, and *Prunus* were occasionally seen; (2) Bushes such as *Ziziphus* and *Tamarix* grew under forests or on ridges and cliffs; (3) Plains may be covered with grasses dominated by *Artemisia*, Compositae, and Chenopodiaceae; and (4) Aquatic or wetland plants such as *Nelumbo*, *Trapa*, Cyperaceae, and *Ceratopteris* were distributed in waters and wetlands. At the same time, artificially managed wild rice growing areas and/or even paddy fields may be present.

The Huai River Basin at this stage presented a relatively similar environmental landscape, which was relatively stable and suitable for human activities. This should be an important basis for the rapid development of Neolithic cultures in the entire region, and it is also an effective guarantee for the development of regional rice farming.

## Rice domestication and cultivation of the Shunshanji Culture

### Rice domestication and crop processing in the Shunshanji Culture

Fluctuating progress during long-term rice domestication was supported by phytolith analyses at numerous sites, including the Shunshanji and Hanjing sites in the mid-lower Huai River, Shangshan, Hehuashan, Kuahuqiao, Tianluoshan and other sites in the Qiantang River Basin (Huan et al., 2014, 2021; Wu et al., 2014; Luo et al., 2016; Zuo et al., 2017; Qiu et al., 2018a). The morphological features of *Oryza*-type bulliform cells, however, point to a discrepancy in the prediction of rice domestication with those of double-peaked *Oryza*-type glume cells (Qiu et al., 2022). The former shows that the rice domestication degree in the mid-lower Huai River is more progressive than that in the Qiantang River. The latter seems to support a contradictory indication.

In addition to studying rice cultivation and domestication, phytolith assemblages can also be used to analyze crop processing (Harvey and Fuller, 2005; Zheng et al., 2009; Weisskopf, 2014; Weisskopf et al., 2014). Moreover, the litter produced by crop processing can also, to some extent, reflect the field system where they are grown and harvested (Weisskopf, 2014; Weisskopf et al., 2014). According to ethnological studies, hulling often occurs near the position where the grain is consumed and the bran is then fed to livestock (Nakai, 2008; Weisskopf et al., 2015a). The vast majority of hulling wastes are generated in the final stages of crop processing, indicating storage of more processed crops and postharvest processing on a larger scale and in larger groups, while primary farm waste (straw, etc.) indicates smaller-scale harvesting and processing, such as smaller household groups (Weisskopf et al., 2015a).

It is to be noted that the concentrations of double-peaked *Oryza*-type glume cells were much higher than those of *Oryza*-type bulliform cells in most samples from the Shunshanji and Hanjing sites during the Shunshanji Culture (Luo et al., 2021; Qiu et al., 2022). This phenomenon implies that rice ears together with the stems and leaves were likely to be stored after the harvest, then threshed and de-husked simultaneously (Luo et al., 2021). The straw and bran by-products were probably tempered in pottery and sintered red soil (Qiu et al., 2022) or discarded around the sites. Meanwhile, it suggests that the Shunshanji Culture presented smaller household groups.

In addition, carbonized rice remains, including rice grain, rice spikelet, depressions of rice husks on pottery sherds and sintered red soil, also shed light on rice domestication and crop processing during the Shunshanji Culture (Luo et al., 2016; Yang et al., 2016; Wu et al., 2017; Qiu et al., 2022).

### Phytolith assemblage indicates paddy cultivation regimes of the Shunshanji Culture

The morphological and chemical characteristics of crops’ phytoliths holds great potential to study ancient irrigation and water conditions in those environments where water is the limiting factor (Madella et al., 2009). In general, rice cultivation regimes include upland rainfed, lowland rainfed or irrigated, flooded or décrue, and deep water cultivation (Weisskopf et al., 2014). Among them, décrue agriculture can be defined as a low-labor input system where seeds were sown into alluvial sedimentary environments after the flood recedes (Harlan and Pasquereau, 1969). The cultivation system can be reflected to a certain extent by the characteristics of the phytolith assemblage. Accordingly, some scholars established the wet/dry model of phytolith assemblage and used it to identify hydration, distinguish millet upland farming from rice farming and differentiate paddy fields from dry fields (Weisskopf, 2017). The paddy field systems of the Tianluoshan, Caoxieshan and Maoshan sites have been analyzed using this model, which illustrates the transformation from submerged paddy fields and drainage fields to large-scale intensive paddy fields (Weisskopf et al., 2015b). Previous studies have shown that the early (8,000–6,000 BP) rice field systems in the mid-lower reaches of the Yangtze River were mostly wetland paddy field systems, which were concentrated in alluvial lowlands and focused on water management, while the early rice cultivation in northern and eastern India was relatively dry monsoon-rainfed system (Fuller and Qin, 2009; Weisskopf et al., 2014). In addition, phytolith combinations (especially those of unidentifiable types) can be used to distinguish cultivated paddy fields from wild rice natural habitats through comparative analysis of modern wild rice growing sites, paddy fields, and non-rice topsoils (Huan et al., 2018, 2020).

The principles of this method are as follows (Blackman, 1968; Piperno, 1988; Madella et al., 2009; Weisskopf et al., 2014; Weisskopf, 2017): (1) Phytoliths in the leaves of herbaceous plants can be divided into fixed or dry form (bilobate, polylobate, long scooped bilobate, rondel, cross, saddle, tall saddle and other short cells) and sensitive or wet morphotype (stomata, elongate psilate, elongate echinate, trapeziform sinuate and other long cells) and (2) The formation of the former type is controlled by genes, while the latter can only be formed by sufficient water absorption. The yield of sensitive morphotypes varies in the whole plant, which is mainly related to the availability of water during plant growth and the effect of general climatic conditions on transpiration (Madella et al., 2009).

**Figure 4:**
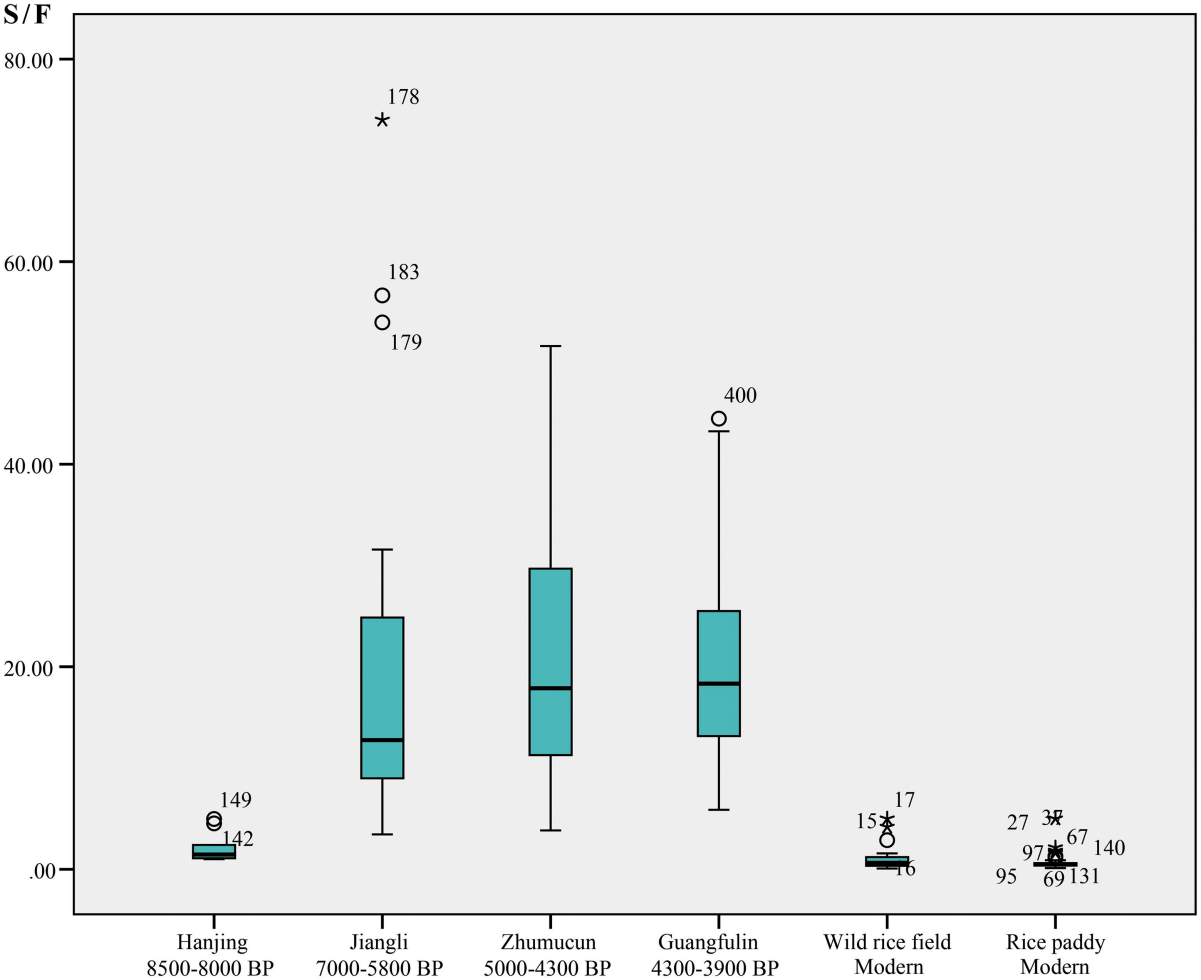
Comparison of phytolith assemblages in paddy fields. The numbers in the figure area indicate the number of anomalous data beyond the edge of the boxplot, where the circles are closer to the edge and the asterisks are further away. The original phytolith data of Hanjing, Jiangli, Zhumucun, Guangfulin, modern wild fields and modern paddy fields was cited from Qiu et al. (2014a,b,c, 2018b, 2022), Zheng (2014), Huan et al. (2018), and Qiu (2021).

Specifically, in the study of ancient paddy fields, the higher content of sensitive forms indicates that rice is growing in a more continuous wet environment, such as the riverside, while more fixed forms indicate drier fields, which were most likely irrigated early and drained late in crop growth (Weisskopf et al., 2015a). This field management operation of irrigation before drainage can increase the yield of many rice varieties (Bhagat et al., 1996). In addition to fixed and sensitive forms, the decrease in Cyperaceae phytoliths and the increase in spongy spicules and diatoms may also indicate better water conditions in paddy fields (Weisskopf et al., 2015a). According to the above analytical principles and methods, the published phytolith assemblages in the paddy fields of Shunshanji (Qiu et al., 2018b, 2022), Majiabang (Qiu et al., 2014b,c), Liangzhu (Qiu et al., 2014a), and Guangfulin (Zheng, 2014) Cultures were integrated, and the corresponding phytolith assemblages in modern paddy fields and wild rice growing fields (Huan et al., 2018; Qiu, 2021) were combined for comparison (Figure 4; Supplementary Table S1). As shown in Figure 4, the S/F ratio of Hanjing (8500–8,000 BP) was between that of the modern wild rice fields and the paddy fields of the Majiabang Culture (7000–5,800 BP), while those between about 7,000–4,000 BP tended to grow with a wider range. The possible reasons could be the variations of the water environment system of ancient paddy fields and the representativeness of the analyzed samples, which need to be further explored by the comparative analysis of paddy fields in the same period.

The ratio of sensitive phytoliths to fixed phytoliths (S/F) suggested that the ancient paddy fields in the mid-lower Huai River and the Taihu Lake Basin, which lasted for more than 4,000 years, seemed to show more persistent and continuous wet conditions (human factors not considered here). Modern paddy fields present the lowest content of fixed forms, which confirms that the long-term farming method of irrigating first and then draining water in relatively dry fields is in line with the actual demand for improving rice yield. Wild rice growing areas tend to be more like lowland rainfed systems, where wild rice is maintained by natural precipitation without human intervention in water supply.

The probable paddy fields of the Shunshanji Culture at the Hanjing site should be in the transition process from a natural lowland system (such as a modern wild rice growing area) to an artificial irrigation and drainage system, such as the ancient paddy fields at the Jiangli site (Qiu et al., 2014b,c), representing the early stage of rice agriculture. This is basically consistent with the results of carbonized plant study and phytolith analysis; that is, during the Shunshanji Culture (8,500–8,000 BP), there may have been a transition from using natural water in lowlands to using wetlands with regular flooding and drainage for rice cultivation (Luo et al., 2021). It also confirmed that rice in the Shunshanji Culture, identified by rice spikelet and phytoliths, was cultivated in the early stage of domestication (Qiu et al., 2018b, 2022).

These two types of paddy field cultivation systems (i.e., natural lowland or wetland system with low-level human interference and artificial irrigation and drainage system) are important representatives of the early development stage of rice farming. They may be the predecessor of the paddy field production system with intensive artificial intervention during the Hemudu and Liangzhu cultures in the lower Yangtze River, represented by the Shi’ao site in Yuyao (Hong, 2020) and the Maoshan site in Linping (Zhao, 2012), respectively. According to the report of China’s Top 10 New Archeological Discoveries of 2021, the total area of the ancient paddy fields at the Shi’ao site in the Ningshao Plain is approximately 900,000 square meters. The three stages of the Shi’ao paddy fields are all characterized by large fields, especially the rice fields of the Liangzhu Culture, which are composed of road networks and irrigation facilities, showing a more complete rice field system. In comparison, the paddy fields in the Liangzhu Culture at the Maoshan site, however, showed more diverse structural and morphological characteristics (Zheng et al., 2009; Zhao, 2012). This is different from the development of the paddy field system from small fields to large fields in the same period around the Taihu Lake area. It is to be noted that many ancient paddy fields were found during the Liangzhu Culture, and their shapes and structures were quite different. Could they represent different paddy farming systems? A comparative study including paddy field morphology, structure and sediment indicators should be carried out in future.

In addition, the rice growing environment revealed by the S/F value could be comparable to the regional environmental landscape. The S/F value of Hanjing indicates a relatively wet lowland rainfed or low-level irrigated system with less human interference in the early Holocene. And that from the Late Neolithic paddy fields at Jiangli, Zhumucun and Guangfulin presents wetter conditions during the Middle Holocene. This is consistent with the results of the Shunshanji Culture revealed by other environmental proxies, such as macro remains of fauna and flora, micro-remains of pollen and phytolith mentioned above as well as the related archeological features unearthed (Qiu et al., 2022).

According to the archeological findings and researches (Nanjing Museum and Sihong Museum, 2016; Zhang, 2018; Zhang et al., 2018), there is no evidence of the direct inheritance relationship between the Shunshanji Culture and the earlier and later archeological cultures in the middle and lower reaches of the Yangtze River. Human migration (ancient DNA irretrievable due to the hostile burial environment) or the spread of early agricultural-related technologies are not observed either. In view of this, we speculate that the mid-lower Huai River is likely to be another independent center for rice cultivation and domestication other than the middle and lower Yangtze region (Qiu et al., 2022). From the above, the important role of the Huai River Basin in the process of prehistoric rice farming is evidenced.

At present, the earliest Neolithic archeological remains in the mid-lower Huai River are from the Shunshanji Culture (*ca.* 8,500–8,000 BP), and its direct cultural origins remains unclear. Regarding its evolution, three archeological cultures in this region, the Shuangdun, Majiabang and Luotuodun cultures, have been discovered, which are about 7,000 years ago and chronologically closest to the Shunshanji Culture (Lin, 2017). However, a big chronological gap of nearly 1,000 years still exists. What’s more, the archeological remains within these cultures were quite different from those in the Shunshanji Culture and an inheritance relationship between them could not been defined. When concentrating on the Shunshanji cultural site group, the excavated sites all presented a third phase (*ca.* 7,800–7,700 BP), which was superimposed directly on the Shunshanji Culture. However, a short interval (*ca.* 8,000–7,800 BP) exists without clear signs of natural deposition or other associated accumulation, which makes it the key to study the evolution of this culture. As for the role of environmental change in the fall of the Shunshanji Culture, no direct research has been conducted yet. Further study is needed and particular attention should be paid on the interval of 8,000–7,800 BP.

## Conclusion

The dispersal of agriculture is influenced by factors such as postdomesticational adaptive genetic changes in plants, anthropogenic changes in agricultural landscapes, and cultural dynamics of food selection (Fuller and Lucas, 2017). It is a good reflection of the effective shaping of human ecosystems and human adaptations. In the early Holocene, various types of rice remains unearthed from the Shunshanji cultural site group revealed that the mid-lower Huai River was in a critical temporal and spatial position for rice resources or the northward expansion of early rice farming. The Shunshanji Culture is at the beginning of the Holocene Megathermal. The regional climate is generally warm, humid and suitable for the development of prehistoric culture, and the local environmental landscape is advantageous.

The sites of Shunshanji Culture show the characteristics of coexistence of homogenization (dominance) and diversification in terms of settlement patterns, cultural factors and subsistence strategies. This enriched the diversity of rice farming in prehistoric China at this stage and shed light on exploring early rice farming systems under different micro-geomorphic environments.

## Author Contributions

ZQ and HR conceptualized and designed the research. ZQ carried out the image process and data analysis. All authors were involved in the writing and discussion of the manuscript, contributed to the article, and approved the final version of the manuscript.

## Funding

This study was supported by the Strategic Priority Research Program of Chinese Academy of Sciences (XDB26000000), the National Natural Science Foundation of China (grant nos. 42107470 and 41702186), and Youth Innovation Promotion Association of CAS (no. 2020076).

## Conflict of interest

The authors declare that the research was conducted in the absence of any commercial or financial relationships that could be construed as a potential conflict of interest.

The handling editor declared a shared affiliation with the author HR at the time of the review. The Reviewer XH declared a shared affiliation with the author HR at the time of the review.

## Publisher’s note

All claims expressed in this article are solely those of the authors and do not necessarily represent those of their affiliated organizations, or those of the publisher, the editors and the reviewers. Any product that may be evaluated in this article, or claim that may be made by its manufacturer, is not guaranteed or endorsed by the publisher.

## Supplementary material

The Supplementary material for this article can be found online at: https://www.frontiersin.org/articles/10.3389/fpls. 2022.966635/full#supplementary-material

## Abbreviations

HPICRA: Henan Provincial Institute of Cultural Relics and Archaeology
CGTN: China Global Television Network
