# Supplementary material for: Environmental landscape and subsistence strategy of the Shunshanji Culture: A review
Source: Front Plant Sci. 2022 Jul 22;13:966635. doi: 10.3389/fpls.2022.966635 (PMC9355314; doi:10.3389/fpls.2022.966635)
Supplement: Supplementary file 1 [file Table_1.DOCX]

Table S1. Assemblage characteristics of sensitive (S) and fixed (F) phytoliths in ancient paddy fields, modern paddy fields and wild rice stands.

| **No.** | **Deposit** | **Sensitive %** | **Fixed %** | **S/F** | **Date yr BP** | **Site** | **E** | **N** | **Reference** |
| --- | --- | --- | --- | --- | --- | --- | --- | --- | --- |
| 1 | T3354g1 | 47.67 | 19.84 | 2.40 | 8500-8000 | Hanjing | 118.22 | 32.59 | Qiu et al. (2018, 2022) |
| 2 | T3354g2 | 58.27 | 11.73 | 4.97 |  |  |  |  |  |
| 3 | T3354g3 | 36.82 | 24.44 | 1.51 |  |  |  |  |  |
| 4 | T3354g4 | 47.47 | 24.90 | 1.91 |  |  |  |  |  |
| 5 | T3354g5 | 27.13 | 27.13 | 1.00 |  |  |  |  |  |
| 6 | T3354g6 | 33.94 | 31.11 | 1.09 |  |  |  |  |  |
| 7 | T3354g7 | 37.33 | 35.20 | 1.06 |  |  |  |  |  |
| 8 | T3354g8 | 39.66 | 28.16 | 1.41 |  |  |  |  |  |
| 9 | T3352k1②-1 | 50.81 | 11.21 | 4.53 |  |  |  |  |  |
| 10 | T3352k1②-2 | 27.25 | 23.11 | 1.18 |  |  |  |  |  |
| 11 | S4(0-2.5) | 33.25 | 2.91 | 11.42 | 7000-5800 | Jiangli | 120.92 | 31.25 | Qiu et al. (2014b, c) |
| 12 | S4(2.5-5) | 38.51 | 4.47 | 8.62 |  |  |  |  |  |
| 13 | S5(0-3) | 31.70 | 1.17 | 27.00 |  |  |  |  |  |
| 14 | S5(3-6) | 29.91 | 3.48 | 8.60 |  |  |  |  |  |
| 15 | S6(0-2) | 43.52 | 4.44 | 9.79 |  |  |  |  |  |
| 16 | S6(2-4) | 45.10 | 6.47 | 6.97 |  |  |  |  |  |
| 17 | S6(4-6) | 50.94 | 3.93 | 12.95 |  |  |  |  |  |
| 18 | S6(6-8) | 41.43 | 2.62 | 15.80 |  |  |  |  |  |
| 19 | S6(8-10) | 37.14 | 4.06 | 9.14 |  |  |  |  |  |
| 20 | S6(10-15) | 25.83 | 2.69 | 9.62 |  |  |  |  |  |
| 21 | S6(15-20) | 38.29 | 3.81 | 10.05 |  |  |  |  |  |
| 22 | S6(20-25) | 37.90 | 3.20 | 11.86 |  |  |  |  |  |
| 23 | S6(25-30) | 42.39 | 2.31 | 18.33 |  |  |  |  |  |
| 24 | S6(30-35) | 51.47 | 4.11 | 12.52 |  |  |  |  |  |
| 25 | S6(35-40) | 50.27 | 1.63 | 30.78 |  |  |  |  |  |
| 26 | S6(40-45) | 54.89 | 2.82 | 19.47 |  |  |  |  |  |
| 27 | S6(45-50) | 47.97 | 1.83 | 26.22 |  |  |  |  |  |
| 28 | S6(50-55) | 35.74 | 4.05 | 8.83 |  |  |  |  |  |
| 29 | S6(55-60) | 29.82 | 2.37 | 12.56 |  |  |  |  |  |
| 30 | S6(60-65) | 37.41 | 4.38 | 8.54 |  |  |  |  |  |
| 31 | S7 | 30.15 | 2.25 | 13.42 |  |  |  |  |  |
| 32 | S8 | 39.11 | 1.81 | 21.56 |  |  |  |  |  |
| 33 | S9 | 28.20 | 1.08 | 26.00 |  |  |  |  |  |
| 34 | S10 | 37.75 | 0.00 |  |  |  |  |  |  |
| 35 | S11 | 41.65 | 0.56 | 74.00 |  |  |  |  |  |
| 36 | S12 | 32.27 | 0.60 | 54.00 |  |  |  |  |  |
| 37 | S13 | 34.16 | 0.00 |  |  |  |  |  |  |
| 38 | S14 | 30.45 | 3.95 | 7.71 |  |  |  |  |  |
| 39 | S15 | 34.16 | 1.44 | 23.71 |  |  |  |  |  |
| 40 | S16 | 31.37 | 0.55 | 56.67 |  |  |  |  |  |
| 41 | S17 | 23.71 | 1.24 | 19.17 |  |  |  |  |  |
| 42 | S18 | 43.76 | 1.39 | 31.57 |  |  |  |  |  |
| 43 | X1 | 36.55 | 10.61 | 3.45 |  |  |  |  |  |
| 44 | X2 | 41.59 | 9.48 | 4.39 |  |  |  |  |  |
| 45 | S1-1 | 49.64 | 1.45 | 34.13 | 5000-4300 | Zhumucun | 120.86 | 31.34 | Qiu et al. (2014a) |
| 46 | S1-2 | 51.90 | 3.62 | 14.33 |  |  |  |  |  |
| 47 | S1-3 | 40.92 | 1.36 | 30.13 |  |  |  |  |  |
| 48 | S2-1 | 53.94 | 2.23 | 24.23 |  |  |  |  |  |
| 49 | S2-2 | 50.85 | 1.53 | 33.33 |  |  |  |  |  |
| 50 | S3-1 | 50.49 | 0.98 | 51.67 |  |  |  |  |  |
| 51 | S3-2 | 53.29 | 4.23 | 12.59 |  |  |  |  |  |
| 52 | S3-3 | 33.22 | 1.20 | 27.57 |  |  |  |  |  |
| 53 | S3-4 | 43.55 | 1.25 | 34.71 |  |  |  |  |  |
| 54 | S4-1 | 44.74 | 1.53 | 29.25 |  |  |  |  |  |
| 55 | S4-2 | 51.64 | 3.96 | 13.04 |  |  |  |  |  |
| 56 | S4-3 | 41.78 | 4.19 | 9.96 |  |  |  |  |  |
| 57 | S5-1 | 18.05 | 4.70 | 3.84 |  |  |  |  |  |
| 58 | S5-2 | 29.45 | 3.44 | 8.56 |  |  |  |  |  |
| 59 | S5-3 | 28.49 | 1.59 | 17.88 |  |  |  |  |  |
| 60 | S5-4 | 23.84 | 1.16 | 20.50 |  |  |  |  |  |
| 61 | S5-5 | 28.57 | 5.42 | 5.27 |  |  |  |  |  |
| 62 | S5-6 | 15.17 | 3.45 | 4.40 |  |  |  |  |  |
| 63 | S5-7 | 24.07 | 1.76 | 13.67 |  |  |  |  |  |
| 64 | 08SGIT3918⑤a-(1) | 63.01 | 3.65 | 17.25 | 4300-3900 | Guangfulin | 121.20 | 31.06 | Zheng (2014) |
| 65 | 08SGIT3918⑤a-(2) | 71.25 | 3.67 | 19.44 |  |  |  |  |  |
| 66 | 08SGIT3918⑤a-(3) | 66.31 | 4.81 | 13.78 |  |  |  |  |  |
| 67 | 08SGIT3918⑤a-(4) | 62.64 | 4.76 | 13.15 |  |  |  |  |  |
| 68 | 08SGIT3918⑤a-(5) | 71.36 | 2.55 | 28.00 |  |  |  |  |  |
| 69 | 08SGIT3918⑤a-(6) | 61.07 | 1.95 | 31.25 |  |  |  |  |  |
| 70 | 08SGIT3918⑤a-(7) | 70.54 | 1.81 | 38.92 |  |  |  |  |  |
| 71 | 08SGIT3918⑤a-(8) | 74.59 | 1.80 | 41.54 |  |  |  |  |  |
| 72 | 08SGIT3918⑤a-(9) | 71.69 | 3.09 | 23.24 |  |  |  |  |  |
| 73 | 08SGIT3918⑤a-(10) | 75.00 | 3.17 | 23.68 |  |  |  |  |  |
| 74 | 08SGIT3918⑤a-(11) | 75.69 | 2.96 | 25.53 |  |  |  |  |  |
| 75 | 08SGIT3918⑤a-(12) | 68.29 | 3.90 | 17.50 |  |  |  |  |  |
| 76 | 08SGIT3918⑤a-(13) | 71.36 | 6.29 | 11.34 |  |  |  |  |  |
| 77 | 08SGIT3918⑤a-(14) | 68.39 | 3.55 | 19.27 |  |  |  |  |  |
| 78 | 08SGIT3918⑤a-(15) | 73.52 | 2.84 | 25.92 |  |  |  |  |  |
| 79 | 08SGIT3918④C-(24)孔 | 67.87 | 6.31 | 10.76 |  |  |  |  |  |
| 80 | 08SGIT3918④C-(11) | 68.41 | 5.44 | 12.58 |  |  |  |  |  |
| 81 | 08SGIT3919④C-(30) | 69.55 | 5.31 | 13.11 |  |  |  |  |  |
| 82 | 08SGIT3918④C-(13) | 70.45 | 3.55 | 19.87 |  |  |  |  |  |
| 83 | 08SGIT3919④C-(28) | 70.16 | 4.44 | 15.82 |  |  |  |  |  |
| 84 | 08SGIT3918④C-(23) | 61.68 | 5.08 | 12.15 |  |  |  |  |  |
| 85 | 08SGIT3919④C-(26) | 71.30 | 2.03 | 35.14 |  |  |  |  |  |
| 86 | 08SGIT3919④C- (31) | 68.06 | 3.44 | 19.79 |  |  |  |  |  |
| 87 | 08SGIT3919④C- (32) | 66.32 | 4.70 | 14.11 |  |  |  |  |  |
| 88 | 08SGIT3918④C-(2) | 64.91 | 2.64 | 24.60 |  |  |  |  |  |
| 89 | 08SGIT3918④C- (15) | 66.44 | 2.91 | 22.85 |  |  |  |  |  |
| 90 | 08SGIT3918④C-(10) | 69.15 | 4.48 | 15.44 |  |  |  |  |  |
| 91 | 08SGIT3918④C- (22) | 63.23 | 2.23 | 28.38 |  |  |  |  |  |
| 92 | 08SGIT3918④C- (16) | 73.62 | 4.91 | 15.00 |  |  |  |  |  |
| 93 | 08SGIT3919④C-(9) | 68.97 | 2.65 | 26.00 |  |  |  |  |  |
| 94 | 08SGIT3918④C- (19) | 62.90 | 6.99 | 9.00 |  |  |  |  |  |
| 95 | 08SGIT3918④C- (20) | 65.32 | 2.68 | 24.33 |  |  |  |  |  |
| 96 | 08SGIT3918④C-(4) | 60.52 | 2.58 | 23.50 |  |  |  |  |  |
| 97 | 08SGIT3918④C-(27) | 64.37 | 6.90 | 9.33 |  |  |  |  |  |
| 98 | 08SGIT3918④C-(9) | 71.34 | 1.65 | 43.25 |  |  |  |  |  |
| 99 | 08SGIT3918④C-(3) | 65.16 | 3.54 | 18.39 |  |  |  |  |  |
| 100 | 08SGIT3918探方-1-(1) | 59.65 | 4.09 | 14.57 |  |  |  |  |  |
| 101 | 08SGIT3918④C-(25) | 64.58 | 8.75 | 7.38 |  |  |  |  |  |
| 102 | 08SGIT3918④C-(12) | 68.05 | 5.29 | 12.86 |  |  |  |  |  |
| 103 | 08SGIT3918④C下中线(18)孔 | 62.62 | 3.13 | 20.00 |  |  |  |  |  |
| 104 | 08SGIT3918④C-(21) | 65.65 | 3.91 | 16.78 |  |  |  |  |  |
| 105 | 08SGIT3918④C-(5) | 72.01 | 4.63 | 15.54 |  |  |  |  |  |
| 106 | 08SGIT3918④C-(14) | 64.35 | 6.62 | 9.71 |  |  |  |  |  |
| 107 | 08SGIT3919④C-(33) | 79.78 | 6.18 | 12.91 |  |  |  |  |  |
| 108 | 08SGIT3918④C-(8) | 62.24 | 1.40 | 44.50 |  |  |  |  |  |
| 109 | 08SGIT3918④C-(17) | 65.90 | 3.61 | 18.27 |  |  |  |  |  |
| 110 | 08SGIT3918④C-(6) | 68.12 | 3.49 | 19.50 |  |  |  |  |  |
| 111 | 08SGIT3918④C-(7) | 57.68 | 4.15 | 13.90 |  |  |  |  |  |
| 112 | 08SGIT3920⑤a-(1)E | 64.71 | 4.12 | 15.71 |  |  |  |  |  |
| 113 | 08SGIT3920⑤a-(2)E | 67.51 | 3.55 | 19.00 |  |  |  |  |  |
| 114 | 08SGIT3920⑤a-LE | 68.22 | 2.33 | 29.33 |  |  |  |  |  |
| 115 | 08SGIT3920⑤a-(1)S | 59.24 | 9.78 | 6.06 |  |  |  |  |  |
| 116 | 08SGIT3920⑤a-(2)S | 62.95 | 10.71 | 5.88 |  |  |  |  |  |
| 117 | 08SGIT3920⑤a-(3) | 62.98 | 4.97 | 12.67 |  |  |  |  |  |
| 118 | 08SGIT0419⑥-H3031 | 72.79 | 4.41 | 16.50 |  |  |  |  |  |
| 119 | 08SGIT0419⑥-H3032 | 80.37 | 1.87 | 43.00 |  |  |  |  |  |
| 120 | 08SGIT0419⑥-H3033 | 78.57 | 1.95 | 40.33 |  |  |  |  |  |
| 121 | 08SGIT0419⑥-H3034 | 80.95 | 3.17 | 25.50 |  |  |  |  |  |
| 122 | 10CL-B1 | 38.73 | 32.15 | 1.20 | Modern | wild rice stands | 113.70 | 26.86 | Huan et al. (2018) |
| 123 | 10CL-B3 | 42.61 | 27.09 | 1.57 |  |  | 113.70 | 26.86 |  |
| 124 | 10CL-B4 | 37.47 | 52.11 | 0.72 |  |  | 113.70 | 26.86 |  |
| 125 | 10CL-B6 | 17.03 | 69.54 | 0.24 |  |  | 113.70 | 26.86 |  |
| 126 | 10CL-B7 | 24.15 | 42.82 | 0.56 |  |  | 113.70 | 26.86 |  |
| 127 | 10CL-B8 | 21.74 | 62.93 | 0.35 |  |  | 113.70 | 26.86 |  |
| 128 | 10CL-B9 | 15.27 | 64.60 | 0.24 |  |  | 113.70 | 26.86 |  |
| 129 | 10CL-S4 | 18.55 | 14.92 | 1.24 |  |  | 113.70 | 26.86 |  |
| 130 | DXA | 25.87 | 20.65 | 1.25 |  |  | 116.53 | 28.11 |  |
| 131 | DXS-1 | 20.19 | 36.25 | 0.56 |  |  | 116.51 | 28.10 |  |
| 132 | DXS-2 | 21.51 | 23.95 | 0.90 |  |  | 116.51 | 28.10 |  |
| 133 | DY-1 | 23.08 | 35.24 | 0.65 |  |  | 116.53 | 28.08 |  |
| 134 | DY-2 | 17.62 | 35.48 | 0.50 |  |  | 116.53 | 28.10 |  |
| 135 | DY-3 | 17.10 | 31.38 | 0.54 |  |  | 116.53 | 28.08 |  |
| 136 | HL-BT1 | 26.26 | 9.13 | 2.87 |  |  | 110.68 | 19.79 |  |
| 137 | HL-BT2 | 22.60 | 5.41 | 4.18 |  |  | 110.68 | 19.79 |  |
| 138 | TS-BT4 | 22.78 | 4.56 | 5.00 |  |  | 110.69 | 19.73 |  |
| 139 | WN-2 | 16.88 | 55.75 | 0.30 |  |  | 110.41 | 18.74 |  |
| 140 | WN-4 | 26.23 | 68.31 | 0.38 |  |  | 110.41 | 18.74 |  |
| 141 | WN-5 | 6.22 | 80.82 | 0.08 |  |  | 110.41 | 18.74 |  |
| 142 | WN-BT6 | 31.59 | 31.34 | 1.01 |  |  | 110.41 | 18.74 |  |
| 143 | WN-BT7 | 12.98 | 79.81 | 0.16 |  |  | 110.41 | 18.74 |  |
| 144 | WN-BT8 | 7.41 | 82.35 | 0.09 |  |  | 110.41 | 18.74 |  |
| 145 | WN-BT9 | 35.54 | 36.03 | 0.99 |  |  | 110.41 | 18.74 |  |
| 146 | ZX-2 | 19.01 | 20.25 | 0.94 |  |  | 110.10 | 18.59 |  |
| 147 | LHT-2 | 12.56 | 20.05 | 0.63 |  |  | 109.50 | 18.23 |  |
| 148 | 10CL | 12.71 | 6.99 | 1.82 | Modern | paddy fields | 113.70 | 26.86 |  |
| 149 | HKML-1 | 13.24 | 6.16 | 2.15 |  |  | 110.50 | 19.92 |  |
| 150 | HL-OS1 | 12.76 | 22.59 | 0.56 |  |  | 110.68 | 19.79 |  |
| 151 | TS-BT1 | 20.09 | 4.02 | 5.00 |  |  | 110.69 | 19.73 |  |
| 152 | TS-BT2 | 16.63 | 35.70 | 0.47 |  |  | 110.69 | 19.73 |  |
| 153 | TS-BT3 | 20.81 | 50.96 | 0.41 |  |  | 110.69 | 19.73 |  |
| 154 | WN-BT1 | 19.71 | 65.32 | 0.30 |  |  | 110.41 | 18.74 |  |
| 155 | WN-BT2 | 13.37 | 37.62 | 0.36 |  |  | 110.41 | 18.74 |  |
| 156 | WN-BT3 | 6.42 | 38.77 | 0.17 |  |  | 110.41 | 18.74 |  |
| 157 | WN-BT4 | 10.77 | 60.29 | 0.18 |  |  | 110.41 | 18.74 |  |
| 158 | DTFJ1 | 43.40 | 26.21 | 1.66 |  |  | 118.21 | 27.63 |  |
| 159 | DTFJ2 | 30.45 | 47.52 | 0.64 |  |  | 117.78 | 27.44 |  |
| 160 | DTFJ3 | 23.04 | 25.79 | 0.89 |  |  | 117.31 | 27.13 |  |
| 161 | DTFJ4 | 24.88 | 66.91 | 0.37 |  |  | 116.75 | 25.80 |  |
| 162 | DTFJ5 | 25.25 | 38.24 | 0.66 |  |  | 116.41 | 25.66 |  |
| 163 | DTFJ6 | 21.87 | 43.98 | 0.50 |  |  | 116.50 | 24.94 |  |
| 164 | DTFJ7 | 16.41 | 28.23 | 0.58 |  |  | 117.75 | 24.73 |  |
| 165 | DTFJ8 | 30.56 | 55.50 | 0.55 |  |  | 118.89 | 25.94 |  |
| 166 | DTFJ9 | 25.58 | 31.57 | 0.81 |  |  | 118.63 | 27.42 |  |
| 167 | DTFJ10 | 15.80 | 51.36 | 0.31 |  |  | 118.86 | 27.35 |  |
| 168 | DTHN1 | 20.67 | 34.67 | 0.60 |  |  | 112.48 | 27.20 |  |
| 169 | DTHN2 | 25.06 | 41.07 | 0.61 |  |  | 112.09 | 27.13 |  |
| 170 | DTHN3 | 16.75 | 37.38 | 0.45 |  |  | 112.22 | 27.51 |  |
| 171 | DTHN4 | 16.35 | 42.65 | 0.38 |  |  | 112.43 | 27.90 |  |
| 172 | DTHN5 | 30.86 | 58.37 | 0.53 |  |  | 112.11 | 27.94 |  |
| 173 | DTHN6 | 14.11 | 63.37 | 0.22 |  |  | 112.42 | 28.20 |  |
| 174 | DTHN7 | 22.44 | 60.43 | 0.37 |  |  | 112.62 | 28.30 |  |
| 175 | DTHN8 | 22.43 | 53.74 | 0.42 |  |  | 112.32 | 28.75 |  |
| 176 | DTHN9 | 20.66 | 32.09 | 0.64 |  |  | 112.51 | 29.02 |  |
| 177 | DTHN10 | 25.57 | 44.98 | 0.57 |  |  | 112.76 | 29.01 |  |
| 178 | DTHN11 | 8.97 | 75.21 | 0.12 |  |  | 111.83 | 28.95 |  |
| 179 | DTHN12 | 23.22 | 54.18 | 0.43 |  |  | 111.10 | 29.57 |  |
| 180 | DTHN13 | 16.63 | 52.42 | 0.32 |  |  | 110.09 | 28.98 |  |
| 181 | DTHN14 | 12.73 | 64.72 | 0.20 |  |  | 112.30 | 25.40 |  |
| 182 | DTHN15 | 14.75 | 34.10 | 0.43 |  |  | 112.56 | 26.38 |  |
| 183 | DTHN16 | 16.41 | 32.83 | 0.50 |  |  | 112.82 | 27.01 |  |
| 184 | DTHN17 | 22.03 | 66.34 | 0.33 |  |  | 112.90 | 27.05 |  |
| 185 | DTHN18 | 29.68 | 48.91 | 0.61 |  |  | 113.03 | 27.08 |  |
| 186 | DTHN19 | 25.48 | 43.74 | 0.58 |  |  | 113.22 | 27.08 |  |
| 187 | DTHN20 | 24.40 | 49.52 | 0.49 |  |  | 113.36 | 26.91 |  |
| 188 | DTHN21 | 26.33 | 23.67 | 1.11 |  |  | 113.53 | 26.82 |  |
| 189 | DTZJ1 | 33.99 | 48.77 | 0.70 |  |  | 119.78 | 30.91 |  |
| 190 | DTZJ2 | 33.48 | 28.04 | 1.19 |  |  | 119.35 | 30.53 |  |
| 191 | DTZJ3 | 33.73 | 42.69 | 0.79 |  |  | 119.63 | 29.84 |  |
| 192 | DTZJ4 | 21.59 | 72.21 | 0.30 |  |  | 119.49 | 29.45 |  |
| 193 | DTZJ5 | 17.08 | 59.00 | 0.29 |  |  | 118.60 | 28.61 |  |
| 194 | DTZJ6 | 28.02 | 32.12 | 0.87 |  |  | 119.02 | 28.93 |  |
| 195 | DTZJ7 | 22.92 | 67.87 | 0.34 |  |  | 121.02 | 28.88 |  |
| 196 | DTZJ8 | 32.48 | 46.26 | 0.70 |  |  | 121.33 | 29.26 |  |
| 197 | DTZJ10 | 36.61 | 56.02 | 0.65 |  |  | 120.65 | 30.55 |  |
| 198 | DTZJ12 | 23.91 | 59.42 | 0.40 |  |  | 118.47 | 28.95 |  |
| 199 | DTZJ13 | 29.68 | 46.88 | 0.63 |  |  | 118.54 | 28.65 |  |
| 200 | DTJX1 | 24.24 | 58.73 | 0.41 |  |  | 114.75 | 26.86 |  |
| 201 | DTJX2 | 19.14 | 40.91 | 0.47 |  |  | 114.39 | 26.79 |  |
| 202 | DTJX3 | 33.25 | 58.13 | 0.57 |  |  | 114.61 | 26.59 |  |
| 203 | DTJX4 | 25.48 | 49.76 | 0.51 |  |  | 114.94 | 26.77 |  |
| 204 | DTJX5 | 19.81 | 55.19 | 0.36 |  |  | 115.29 | 26.30 |  |
| 205 | DTJX6 | 16.06 | 54.50 | 0.29 |  |  | 114.94 | 25.35 |  |
| 206 | DTJX7 | 17.88 | 38.63 | 0.46 |  |  | 115.72 | 25.48 |  |
| 207 | DTJX8 | 28.09 | 49.79 | 0.56 |  |  | 116.52 | 27.36 |  |
| 208 | DTJX9 | 25.34 | 53.88 | 0.47 |  |  | 116.18 | 27.82 |  |
| 209 | DTJX10 | 24.20 | 39.51 | 0.61 |  |  | 115.89 | 28.10 |  |
| 210 | DTJX11 | 14.75 | 75.64 | 0.20 |  |  | 115.66 | 28.37 |  |
| 211 | DTJX12 | 32.16 | 59.03 | 0.54 |  |  | 117.65 | 28.49 |  |
| 212 | DTJX13 | 23.78 | 58.28 | 0.41 |  |  | 116.92 | 28.42 |  |
| 213 | DTJX14 | 21.69 | 47.23 | 0.46 |  |  | 116.94 | 28.89 |  |
| 214 | DTJX15 | 21.86 | 37.91 | 0.58 |  |  | 116.78 | 29.16 |  |
| 215 | DTJX16 | 22.18 | 31.42 | 0.71 |  |  | 115.90 | 29.01 |  |
| 216 | DTJX17 | 33.33 | 26.10 | 1.28 |  |  | 116.13 | 28.98 |  |
| 217 | DTJX18 | 26.97 | 38.19 | 0.71 |  |  | 114.97 | 28.21 |  |
| 218 | DTJX19 | 25.17 | 15.81 | 1.59 |  |  | 114.16 | 27.72 |  |
| 219 | JX-1 | 28.57 | 61.33 | 0.47 |  |  | 116.53 | 28.12 |  |
| 220 | JX-2 | 20.71 | 48.55 | 0.43 |  |  | 116.78 | 28.25 |  |
| 221 | JX-3 | 21.48 | 42.73 | 0.50 |  |  | 116.28 | 28.33 |  |
| 222 | JX-4 | 23.27 | 61.88 | 0.38 |  |  | 115.13 | 26.15 |  |
| 223 | JX-5 | 21.32 | 60.54 | 0.35 |  |  | 115.13 | 26.15 |  |
| 224 | JX-6 | 25.00 | 69.14 | 0.36 |  |  | 115.90 | 28.40 |  |
| 225 | JX-7 | 25.45 | 66.36 | 0.38 |  |  | 115.83 | 28.22 |  |
| 226 | JX-8 | 28.01 | 63.88 | 0.44 |  |  | 115.52 | 28.08 |  |
| 227 | JX-9 | 18.31 | 50.36 | 0.36 |  |  | 115.25 | 27.82 |  |
| 228 | JX-10 | 18.01 | 52.66 | 0.34 |  |  | 115.12 | 27.58 |  |
| 229 | JX-11 | 24.10 | 62.17 | 0.39 |  |  | 115.02 | 27.30 |  |
| 230 | JX-12 | 23.23 | 57.42 | 0.40 |  |  | 114.87 | 27.00 |  |
| 231 | JX-13 | 22.60 | 71.87 | 0.31 |  |  | 114.90 | 26.85 |  |
| 232 | JX-14 | 22.17 | 52.46 | 0.42 |  |  | 115.27 | 26.48 |  |
| 233 | JX-15 | 24.64 | 62.32 | 0.40 |  |  | 115.85 | 26.37 |  |
| 234 | JX-16 | 17.83 | 40.24 | 0.44 |  |  | 116.30 | 26.43 |  |
| 235 | JX-17 | 20.75 | 72.73 | 0.29 |  |  | 116.33 | 26.72 |  |
| 236 | JX-18 | 23.52 | 63.70 | 0.37 |  |  | 116.35 | 26.88 |  |
| 237 | JX-19 | 17.36 | 55.75 | 0.31 |  |  | 116.62 | 27.57 |  |
| 238 | JX-20 | 22.73 | 61.69 | 0.37 |  |  | 116.25 | 28.12 |  |
| 239 | W-P-1 | 16.93 | 39.79 | 0.43 |  |  | 120.12 | 31.52 | Qiu (2021) |
| 240 | W-P-3 | 24.57 | 47.79 | 0.51 |  |  |  |  |  |
| 241 | W-P-5 | 21.53 | 45.21 | 0.48 |  |  |  |  |  |
| 242 | W-P-7 | 15.36 | 41.95 | 0.37 |  |  |  |  |  |
| 243 | W-P-9 | 13.44 | 24.18 | 0.56 |  |  |  |  |  |
| 244 | W-P-11 | 19.37 | 29.05 | 0.67 |  |  |  |  |  |
| 245 | W-P-13 | 15.89 | 43.18 | 0.37 |  |  |  |  |  |
| 246 | W-P-15 | 15.85 | 36.07 | 0.44 |  |  |  |  |  |
| 247 | W-P-17 | 9.01 | 51.65 | 0.17 |  |  |  |  |  |
| 248 | W-P-19 | 12.05 | 45.76 | 0.26 |  |  |  |  |  |
| 249 | W-P-21 | 13.79 | 21.46 | 0.64 |  |  |  |  |  |
| 250 | W-P-23 | 18.43 | 26.30 | 0.70 |  |  |  |  |  |
| 251 | W-P-25 | 14.29 | 35.62 | 0.40 |  |  |  |  |  |
| 252 | W-P-27 | 23.56 | 18.20 | 1.29 |  |  |  |  |  |
| 253 | W-UP-29 | 14.78 | 30.13 | 0.49 |  |  |  |  |  |
| 254 | W-UP-31 | 9.20 | 29.60 | 0.31 |  |  |  |  |  |
| 255 | W-UP-33 | 14.95 | 28.54 | 0.52 |  |  |  |  |  |
| 256 | W-UP-35 | 15.16 | 23.84 | 0.64 |  |  |  |  |  |
| 257 | W-UP-37 | 15.40 | 25.93 | 0.59 |  |  |  |  |  |
| 258 | W-UP-39 | 15.27 | 27.86 | 0.55 |  |  |  |  |  |
| 259 | W-UP-41 | 18.75 | 24.22 | 0.77 |  |  |  |  |  |
| 260 | W-UP-43 | 14.51 | 51.57 | 0.28 |  |  |  |  |  |
| 261 | W-UP-45 | 21.38 | 13.01 | 1.64 |  |  |  |  |  |

**References**

Huan, X., Lu, H., Zhang, J., and Wang, C. (2018). Phytolith assemblage analysis for the identification of rice paddy. *Scientific Reports* 8(1)**,** 10932. doi: 10.1038/s41598-018-29172-5.

Qiu, Z. (2021). Analysis of micro plant remains from modern paddy field. *Agricultural Archaeology* (3), 7-13 (in Chinese with English abstract).

Qiu, Z., Ding, J., Jiang, H., and Hu, Y. (2014a). Analysis on plant remains from rice paddy fields of the Liangzhu Culture at the Zhumucun site, Kunshan City. *Southeast Culture* (2), 57-67 (in Chinese with English abstract).

Qiu, Z., Jiang, H., Ding, J., Hu, Y., and Rao, H. (2014b). Phytolith analysis of paddy fields of Majiabang Culture at Jiangli Site, Kunshan City, Jiangsu. *Oriental Archaeology* 11, 374-386 (in Chinese with English abstract).

Qiu, Z., Jiang, H., Ding, J., Hu, Y., and Shang, X. (2014c). Pollen and phytolith evidence for rice cultivation and vegetation change during the Mid-Late Holocene at the Jiangli site, Suzhou, East China. *PLoS One* 9(1)**,** e86816. doi: 10.1371/journal.pone.0086816.

Qiu, Z., Zhuang, L., and Lin, L. (2018). Phytolith evidence for rice domestication of the Hanjing site in Sihong, Jiangsu Province and the related issues. *Southeast Culture* (1)**,** 71-80 (in Chinese with English abstract).

Qiu, Z., Zhuang, L., Rao, H., Lin, L., and Zhuang, Y. (2022). Excavation at Hanjing site yields evidence of early rice cultivation in the Huai River more than 8000 years ago. *Science China Earth Sciences* 65(5)**,** 910-920. doi: 10.1007/s11430-021-9885-x.

Zheng, X. (2014). *The Research of Prehistoric Paddy in Jiaodong Region*. Dissertation for Master’s Degree, Shandong University (in Chinese with English abstract).
